# *DHX36*, *BAX*, and *ARPC1B* May Be Critical for the Diagnosis and Treatment of Tuberculosis

**DOI:** 10.1155/2020/4348371

**Published:** 2020-07-23

**Authors:** Yunli Zhang, Yanming Li, Hongling Li, Qingxia Liu, Wei Wang, Zijuan Jian, Wenen Liu

**Affiliations:** Department of Clinical Laboratory, Xiangya Hospital of Central South University, Changsha 410008, Hunan, China

## Abstract

**Background:**

Tuberculosis (TB) is usually caused by *Mycobacterium tuberculosis*, which has the highest mortality rate among infectious diseases. This study is designed to identify the key genes affecting the diagnosis and treatment of TB.

**Methods:**

GSE54992, which included 39 peripheral blood mononuclear cell (PBMC) samples, was extracted from the Gene Expression Omnibus database. After the samples were classified into type and time groups by limma package, the differentially expressed genes (DEGs) were analyzed using the Analysis of Variance. Using pheatmap package, hierarchical cluster analysis was performed for the DEGs. Then, the key modules correlated with TB were selected using the WGCNA package. Finally, functional and pathway enrichment analyses were carried out using clusterProfiler package.

**Results:**

The DEGs in subclusters 3, 6, 7, and 8 were chosen for further analyses. Based on WGCNA analysis, blue and green modules in type group and pink module in time group were selected as key modules. From the key modules, 9 (including *BAX* and *ARPC1B*) hub genes in type group and 6 (including *DHX36*) hub genes in time group were screened. Through pathway enrichment analysis, the TNF signaling pathway was enriched for the green module.

**Conclusion:**

*DHX36*, *BAX*, and *ARPC1B* might be key genes acting in the mechanisms of TB. Besides, the TNF signaling pathway might also be critical for the diagnosis and therapy of the disease.

## 1. Introduction

As an infectious disease, tuberculosis (TB) is mainly induced by *Mycobacterium tuberculosis* (MTB) and usually affects the lungs [1, 2]. Latent TB has no obvious signs, and approximately 10% of them can develop into active disease [3]. The typical symptoms of active TB include night sweats, coughing blood, weight loss, and fever [4]. Latent TB is not spread, but active TB can be spread via the air when lung TB patients spit, cough, sneeze, or speak [5]. Smokers and people with human immunodeficiency virus (HIV)/Acquired Immune Deficiency Syndrome (AIDS) are at high risk of active TB, and thus early screening and treatment of high-risk people and vaccination are the main methods for preventing TB [6–8]. A third of the world's population suffers from TB, and the mortality of TB ranks first among infectious diseases [9]. Active TB affects over 10 million people and leads to 1.3 million death cases in 2016 [10, 11]. Therefore, TB should be deeply investigated to reveal its mechanisms.

The product of the Intracellular Pathogen Resistance 1 (*IPR1*) gene may function in integrating signals induced by intracellular pathogens through mediating cell death, innate immunity, and pathogenesis, and thus *IPR1* is a candidate gene controlling the host resistance to TB [12]. Murine *β*-defensin–3 (*mBD3*) and *mBD4* expression are induced by mycobacterial infection, which may play roles in controlling mycobacterial growth during TB infection [13]. As an inner membrane transporter of MTB, transmembrane transport protein MmpL3 (*MMPL3*) participants in the transport of trehalose monomycolate and is a novel target for the treatment of TB patients [14]. Programmed death 1 (*PD-1*) is implicated in the functions of T cell effector against MTB; therefore, *PD-1* can mediate the immune response in hosts during human TB [15, 16]. Through the interleukin (IL) 4R*α* signaling pathway, the T helper (Th) 2 response regulates the alternative activation of macrophages and thus contributes to the intracellular persistence of MTB [17]. However, the above researches only report a part of the genes involved in TB, and more studies should be conducted to fully reveal the pathogenesis of the disease.

In 2014, Cai et al. determine the expression pattern of *C1q* in peripheral blood mononuclear cells (PBMCs) to explore the function of *C1q* in TB, finding that *C1q* is closely related to the active disease and disease severity in TB and serves as a diagnostic biomarker for the disease [18]. Nevertheless, more genes correlated with the diagnosis and progression of TB needed to be explored to prevent the deterioration of TB. Through performing comprehensive bioinformatics analyses for the microarray dataset uploaded by Cai et al. [18], the genes playing key roles in the progression of TB were investigated. This study might broaden our understanding of the mechanisms of TB and promote the diagnosis and treatment of the disease.

## 2. Materials and Methods

### 2.1. Data Source

The expression profiling data of TB (accession number: GSE54992), which was based on the platform of GPL570 [HG-U133_Plus_2] Affymetrix Human Genome U133 Plus 2.0 Array, was extracted from Gene Expression Omnibus (GEO, http://www.ncbi.nlm.nih.gov/geo/) database. There were a total of 39 PBMC samples in GSE54992, including 6 samples from healthy donors (HD), 6 samples from patients with latent TB infection (LTBI), 9 samples from TB patients (TB), 9 samples from TB patients after antituberculosis treatment for 3 months (TB3m), and 9 samples from TB patients after antituberculosis treatment for 6 months (TB6m). The samples were from participants recruited at Shenzhen Third People's Hospital from May 2011 to December 2012. PBMCs were separated from heparinized whole blood as described previously [19]. Cai et al. [18] uploaded GSE54992, and their research was approved by the Institutional Review Board of Shenzhen Third People's Hospital and obtained the informed consent of all participants.

### 2.2. Data Preprocessing and Differential Expression Analysis

The original data in GSE54992 were preprocessed using the R packages Affy [20, 21] (version 1.52.0, http://bioconductor.org/packages/release/bioc/html/affy.html) and limma [22] (version 3.32.2, https://bioconductor.org/packages/release/bioc/html/limma.html). The preprocessing processes were background correction, normalization, log2 conversion, and probe annotation. The probes without matching gene symbols were filtered out. Afterward, the expression value of the gene corresponding to several probes was acquired by calculating the mean value of the probes.

Using the R package limma [22], the data were conducted with standardization analysis, and the samples were classified into type groups (including HD, LTBI, and TB samples) and time groups (including TB, TB3m, and TB6m samples). Based on the Analysis of Variance (ANOVA) [23], the differentially expressed genes (DEGs) between type and time groups were analyzed. The DEGs were defined as genes with *p* value ≤ 0.05 and |fold change (FC)| ≥ 2.

### 2.3. Hierarchical Cluster Analysis

To identify the genes with similar expression patterns, hierarchical cluster analysis was performed for the DEGs using the R package pheatmap [24] (version 1.0.2, https://cran.r-project.org/web/packages/pheatmap/index.html). The distance calculation algorithm, genetic clustering method, and the clustering method for gene clusters separately were Euclidean, kmeans, and hcluster. To screen the targets that could be used for the diagnosis and treatment of TB, the genes significantly dysregulated between TB and HD/LTBI groups and that were near the expression in HD group along with TB-TB3m-TB6m treatments were defined as TB-specific genes and utilized for the subsequent analyses.

### 2.4. Weighted Gene Coexpression Network Analysis (WGCNA)

WGCNA is an algorithm developed for investigating module information from high-throughput data [25]. The R package WGCNA (version 1.61, https://cran.r-project.org/web/packages/WGCNA/index.html) [25] was applied for analyzing the DEGs and the expression data of the DEGs were utilized as the input data for building the coexpression network. The main processes of WGCNA were coexpression network construction and module identification, the identification of disease-associated modules, enrichment analysis for key modules and protein network construction, and the identification and enrichment analysis of hub nodes in the key modules.

### 2.5. Functional and Pathway Enrichment Analysis

Using the R package clusterProfiler (version 3.4.4, https://bioconductor.org/packages/release/bioc/html/clusterProfiler.html) [26], Gene Ontology (GO) [27] functional and Kyoto Encyclopedia of Genes and Genomes (KEGG) [28] pathway enrichment analyses for the DEGs were conducted. The Benjamini and Hochberg (BH) method [29] was used for adjusting the *p* values, and the adjusted *p* value <0.01 was set as the threshold for significant results.

## 3. Results

### 3.1. Differential Expression Analysis

**Figure 1:**
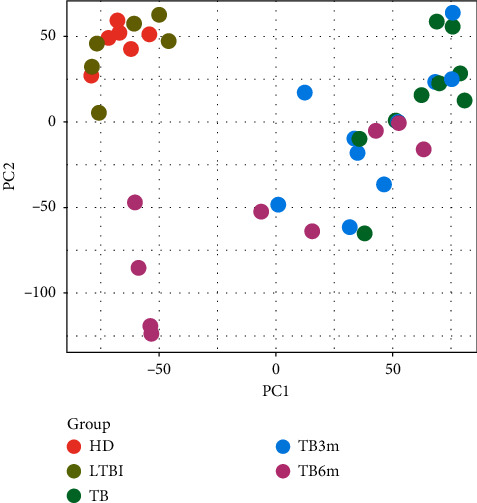
Principal component analysis (PCA) for the samples from healthy donors (HD), the samples from patients with latent TB infection (LTBI), the samples from tuberculosis (TB) patients (TB), the samples from TB patients after antituberculosis treatment for 3 months (TB3m), and the samples from TB patients after antituberculosis treatment for 6 months (TB6m).

After the 54676 probes in GSE54992 were preprocessed, 23520 genes were obtained. Principal component analysis (PCA) for the samples showed that HD and LTBI samples had little differences, and TB and TB3m samples had similar expression patterns (Figure 1).

**Figure 2:**
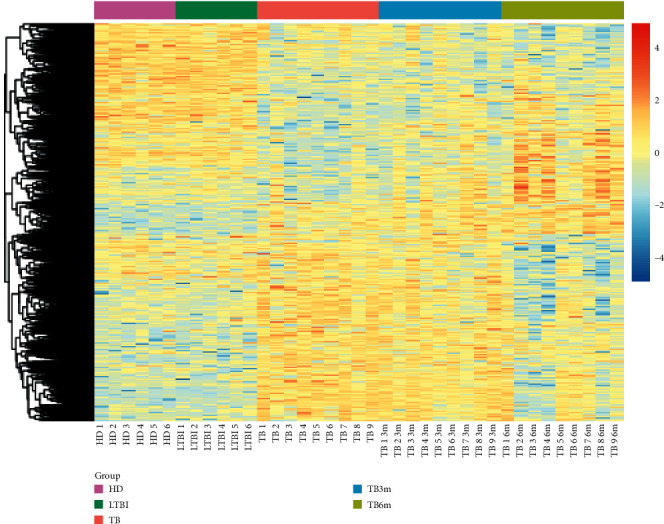
The heatmap showing that the differentially expressed genes (DEGs) can well separate the samples from healthy donors (HD), the samples from patients with latent tuberculosis (TB) infection (LTBI), the samples from TB patients (TB), the samples from TB patients after antituberculosis treatment for 3 months (TB3m), and the samples from TB patients after antituberculosis treatment for 6 months (TB6m). Red and blue represent high and low expressions, respectively.

There were 520, 2931, and 2887 DEGs separately in LTBI vs. HD, TB vs. HD, and TB vs. LTBI type comparison groups. Besides, a total of 462, 1502, and 741 DEGs separately were screened in TB3m vs. TB, TB6m vs. TB, and TB6m vs. TB3m time comparison groups (Table 1). The cluster heatmap for all DEGs showed that the DEGs could separate the samples in different groups very well (Figure 2).

### 3.2. Hierarchical Cluster Analysis

**Figure 3:**
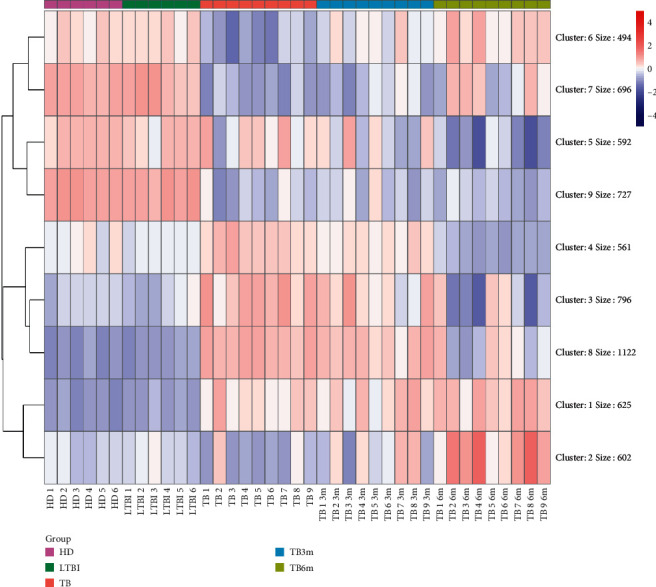
Hierarchical cluster analysis for the differentially expressed genes (DEGs). HD, healthy donors; LTBI, patients with latent tuberculosis (TB) infection; TB, tuberculosis patients; TB3m, tuberculosis patients after antituberculosis treatment for 3 months; TB6m, tuberculosis patients after antituberculosis treatment for 6 months.

With kmeans = 9, the DEGs were performed with hierarchical cluster analysis (Figure 3). Subsequently, the genes in each cluster were compared, and the genes (a total of 3108 genes) in subclusters 3, 6, 7, and 8 were chosen for the following analyses.

### 3.3. WGCNA Analysis

**Figure 4:**
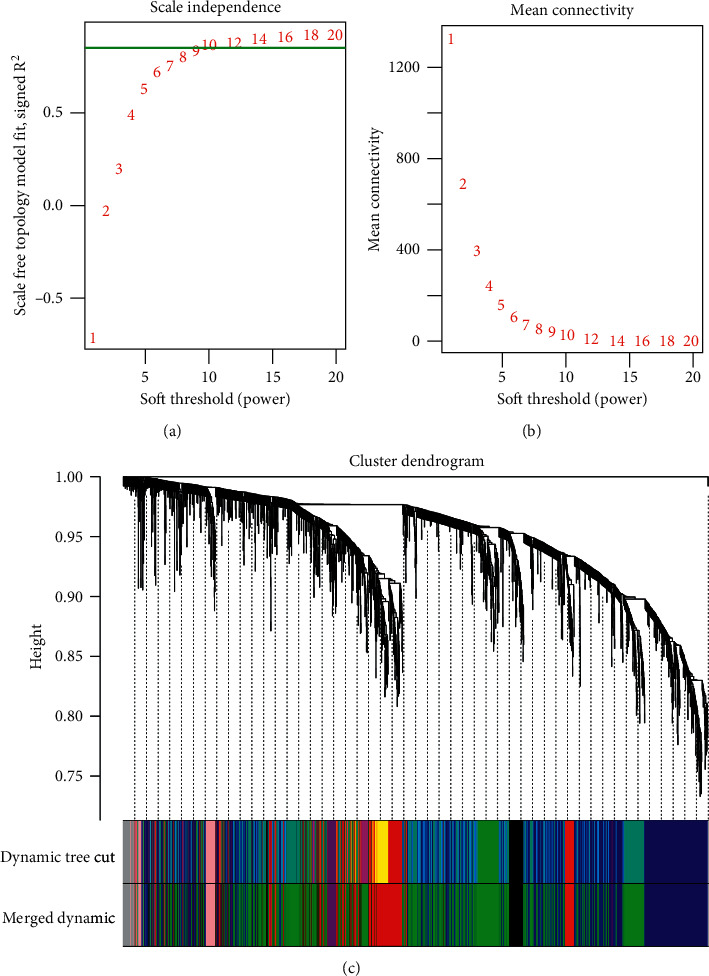
The selection of the weighting parameter *β* (soft threshold power) (the green line indicates that the square approached 0.85) (a), the mean connectivity of genes under different *β* values (b), and the system cluster tree for separating modules (modules are shown as different colors, and the grey module is a set of genes that cannot be clustered into other modules) (c).

The expression matrixes of the 3108 DEGs in the key subclusters were extracted and taken as the input data for constructing coexpression network. Coexpression network should have the characteristics of a scale-free network. Therefore, the weighting parameter *β* (soft threshold power) needed to be negatively correlated with the square of the correlation coefficients between log (*k*) and log (*p*(*k*)). The higher the square was, the closer the coexpression network was to scale-free network. When the square firstly approached 0.85, the corresponding *β* = 10 was suitable for building coexpression network (Figure 4(a)). Besides, the mean connectivity was −1.35 when the *β* value was 10 (Figure 4(b)).

After the system clustering tree was obtained for the genes, 12 network modules (at least 30 genes were involved in each module) were identified (Figure 4(c)). Then, cluster analysis for the modules was conducted, and a total of 8 modules (black module, involving 82 genes; blue module, involving 953 genes; brown module, involving 473 genes; green module, involving 1267 genes; grey module, involving 103 genes; pink module, involving 75 genes; purple module, involving 47 genes; red module, involving 108 genes) were finally obtained after merging the closely clustered modules (height was set at 0.1) (Figure 4(c)).

**Figure 5:**
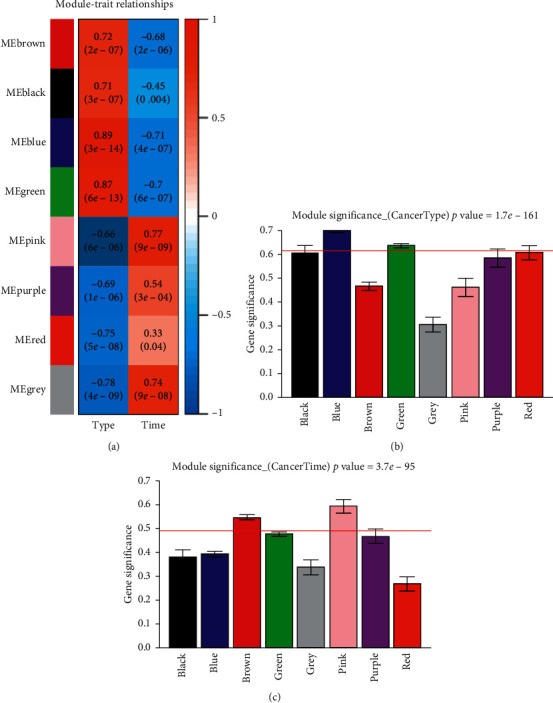
The correlation coefficient between each module and disease state (a), the gene significance (GS) for the modules in type group (b), and the GS for the modules in time group (c).

Gene significance (GS) is defined as the mediated *p* value of each gene in the linear regression between gene expression and the sample traits. Module significance (MS) was defined as the average GS within modules and was calculated to measure the correlation between modules and sample traits. If GS and MS are highly correlated, it means that genes are the most important elements of modules and are highly significantly associated with the trait. According to the absolute value of the correlation coefficient between each module and disease state, blue, green, and red modules were the top 3 modules in the type group. Meanwhile, pink, blue, and green modules were the top 3 modules in the time group (Figure 5(a)). Based on the absolute value of the GS in each module, the key modules in type (blue and green modules) (Figure 5(b)) and time (brown and pink modules) (Figure 5(c)) groups separately were selected. As a result, blue and green modules in type group and pink module in time group were selected by both of the two methods and thus used for screening the hub genes related to the disease.

**Figure 6:**
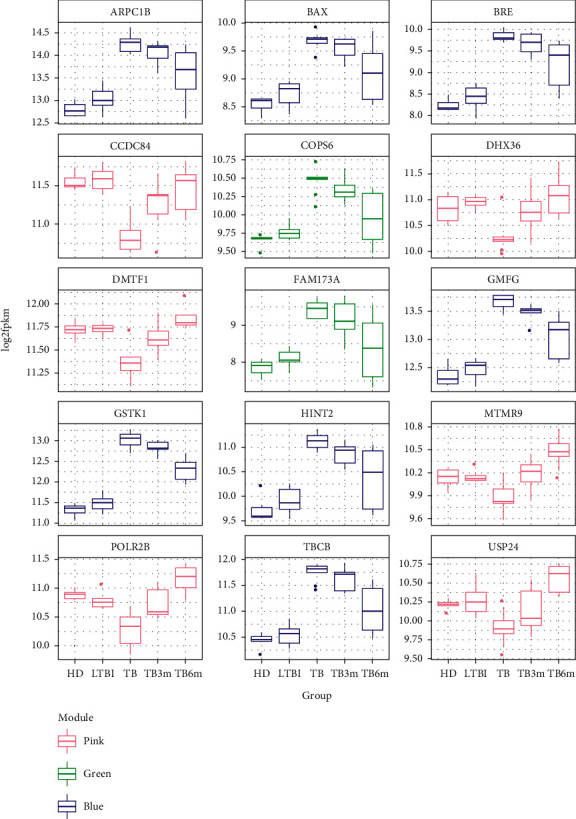
The expression diagrams of the 15 hub genes (ARPC1B, BAX, BRE, CCDC84, COPS6, DHX36, DMTF1, FAM173A, GMFG, GSTK1, HINT2, MTMR9, POLR2B, TBCB, and USP24). HD, healthy donors; LTBI, patients with latent tuberculosis (TB) infection; TB, tuberculosis patients; TB3m, tuberculosis patients after antituberculosis treatment for 3 months; TB6m, tuberculosis patients after antituberculosis treatment for 6 months.

There separately were 874 and 27 hub nodes in type and time groups with the cutoff of MS > 0.8 (*p* value < 0.01) and GS > 0.2 (*p* value < 0.01). Besides, the top 10 genes were selected as candidate hub genes with networkScreening function in the WGCNA package. In addition, the hub nodes were intersected with these candidate hub genes, and the overlapped genes were redefined as the hub genes. Finally, 9 hub genes (including BCL2-associated X protein, *BAX*; and Actin-Related Protein 2/3 Complex, Subunit 1B, *ARPC1B*) in type group and 6 hub genes (including DEAH (Asp-Glu-Ala-His) box polypeptide 36, *DHX36*) in time group were screened. The expression diagrams of the 15 hub genes are shown in Figure 6. The 15 hub genes were mainly involved in pink and blue modules, among which 6 hub genes in time group were specifically downregulated expressed in TB, and 9 hub genes in type group were specifically upregulated expressed in TB.

### 3.4. Enrichment Analysis for Key Modules and Hub Genes

**Figure 7:**
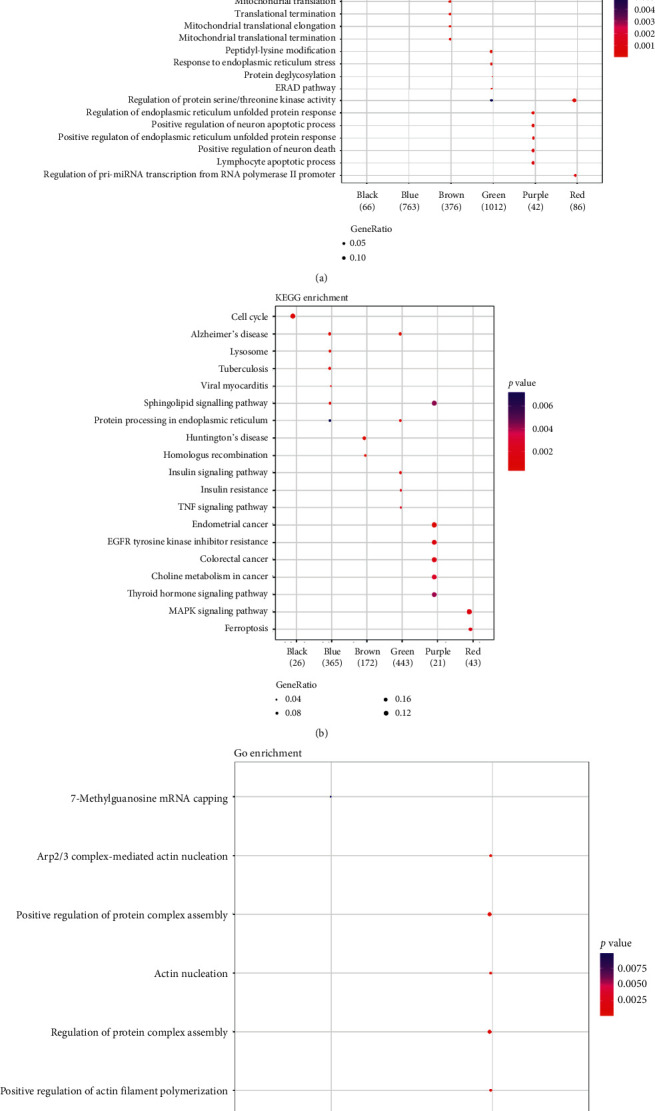
The functional terms enriched for the key modules (a), the pathways enriched for the key modules (b), and the functional terms enriched for the hub genes in type and time groups (c). GO, Gene Ontology; KEGG, Kyoto Encyclopedia of Genes and Genomes.

Functional (Figure 7(a)) and pathway (Figure 7(b)) enrichment analyses for the key modules showed that no significant functional term and pathway were enriched for the pink module. Besides, the tumor necrosis factor (TNF) signaling pathway was enriched for the green module. Moreover, the hub genes in type and time groups were also conducted with enrichment analysis. The results showed that the hub genes in the time group were implicated in the functional term of 7-methylguanosine mRNA capping (Figure 7(c)). However, the hub genes had no significantly enriched pathways.

## 4. Discussion

In this study, the DEGs in type and time comparison groups separately were screened. After performing hierarchical cluster analysis, the DEGs in subclusters 3, 6, 7, and 8 were chosen for further analyses. WGCNA analysis indicated that blue and green modules in the type group and pink module in the time group were key modules. Subsequently, 9 (including *BAX* and *ARPC1B*) and 6 (including *DHX36*) hub genes separately were identified in type group and time group. Pathway enrichment analysis showed that the TNF signaling pathway was enriched for the green module.

Increased B-cell CLL/lymphoma 2 (*BCL2*) and decreased *BAX* are detected in macrophages, and *BCL2* overexpression in macrophages carrying MTB may be related to its intracellular survival [30]. Immunohistochemical staining shows that overexpressed *BAX*, *P53*, and Fas cell surface death receptor (*FAS*) have correlations with reduced *BCL2* in TB granulomas [31]. MTB infection causes apoptosis of human neutrophils through inducing reactive oxygen species- (ROS-) dependent expression change of Bax/Bcl-x(L) and caspase-3 activation [32]. The recombinant Bacille Calmette-Guerin (rBCG): BAX strain contributes to the induction of Th1 protective immune responses, which may be a promising vaccine candidate for TB [33]. *ARPC1B* deficiency can lead to severe combined immunodeficiency with signs of mild bleeding and immune disorder [34]. A previous study considers that *DHX36* is correlated with the DNA biosensor of MTB [35]. These indicated that *DHX36*, *BAX*, and *ARPC1B* might be related to the mechanisms of TB.

Proteinase-activated receptor-2 (PAR2), TNF, and galectin 9 (GAL9) pathways are essential for limiting MTB growth, and lipoarabinomannan (LAM) can reduce their activation to promote the intracellular growth of MTB [36]. The interactions between MTB grown in the condition of hypoxia and host macrophages can induce the TNF signaling pathway, DNA-damage stress response, and activation of apoptosis, especially, MTB-H bacilli which are sensitive to TNF-governed killing [37, 38]. *TNF* plays a role in MTB-induced macrophage apoptosis, which is correlated with the TNF- and c-Cbl-dependent FLIP(S)-degradation pathway [39]. Thus, the genes in the green module might function in TB via the TNF signaling pathway.

In conclusion, *DHX36*, *BAX*, and *ARPC1B* might be involved in the diagnosis and treatment of TB. In addition, the TNF signaling pathway might also be important for the development of TB. However, experimental researches should be carried out in the future to support our results.

## Figures and Tables

**Table 1:** The number of differentially expressed genes (DEGs) identified in each comparison group. HD, healthy donors; LTBI, patients with latent tuberculosis (TB) infection; TB, tuberculosis patients; TB3m, tuberculosis patients after antituberculosis treatment for 3 months; TB6m, tuberculosis patients after antituberculosis treatment for 6 months.

Comparison groups	Number of DEGs	Number of upregulated genes	Number of downregulated genes
LTBI vs. HD	520	201	319
TB vs. HD	2931	1488	1443
TB vs. LTBI	2887	1505	1382
TB3m vs. TB	462	249	213
TB6m vs. TB	1502	395	1107
TB6m vs. TB3m	741	170	571

## Data Availability

The datasets analyzed for this study can be found in the Gene Expression Omnibus (GEO) database (GSE54992) (http://www.ncbi.nlm.nih.gov/geo/).

## Abbreviations

PCA: Principal component analysis
HD: Healthy donors
LTBI: Latent TB infection
TB: Tuberculosis
DEGs: Differentially expressed genes
MTB: Mycobacterium tuberculosis
HIV: Human immunodeficiency virus
AIDS: Acquired Immune Deficiency Syndrome
*IPR1*: Intracellular Pathogen Resistance 1
PBMCs: Peripheral blood mononuclear cells
TNF: Tumor necrosis factor
LAM: Lipoarabinomannan.

## References

[1] Kim P. (2009). Tuberculosis, *Mycobacterium tuberculosis*. *Journal of Medicinal Chemistry*.

[2] Chen M., Deng J., Li W. (2015). Impact of tea drinking upon tuberculosis: a neglected issue. *BMC Public Health*.

[3] Lin P. L., Flynn J. L. (2010). Understanding latent tuberculosis: a moving target. *The Journal of Immunology*.

[4] Mandell G., Bennett J., Dolin R. (2005). *Mandell, Douglas, and Bennett’s Principles and Practice of Infectious Diseases*.

[5] Herrera M., Bosch P., Nájera M., Aguilera X. (2013). Modeling the spread of tuberculosis in semiclosed communities. *Computational & Mathematical Methods in Medicine*.

[6] Xie X., Chen J.-w., Li F., Tian J., Gao J.-s., Zhang D. (2011). A T-cell-based enzyme-linked immunospot assay for tuberculosis screening in Chinese patients with rheumatic diseases receiving infliximab therapy. *Clinical and Experimental Medicine*.

[7] Abuaku B., Tan H., Li X., Chen M., Huang X. (2010). Treatment default and death among tuberculosis patients in Hunan, China. *Scandinavian Journal of Infectious Diseases*.

[8] Abuaku B. K., Tan H., Li X., Chen M., Huang X. (2010). A comparative analysis of tuberculosis treatment success between Hunan Province of China and Eastern Ghana. *Medical Principles and Practice*.

[9] Floyd K., Glaziou P., Sismanidis C., Raviglione M. (2013). Global epidemiology of tuberculosis. *Seminars in Respiratory & Critical Care Medicine*.

[10] Organization W. H. (2016). Global tuberculosis report 2016. *Global Tuberculosis Report*.

[11] Chen M., Kwaku A., Chen Y., Huang X., Tan H., Wen Shi (2014). Gender and regional disparities of tuberculosis in Hunan, China. *International Journal for Equity in Health*.

[12] Pan H., Yan B.-S., Rojas M. (2005). Ipr1 gene mediates innate immunity to tuberculosis. *Nature*.

[13] Rivassantiago B., Sada E., Tsutsumi V., Aguilar-Leon D., Leon Contreras J., Hernandez-Pando R. (2006). *β*-Defensin gene expression during the course of experimental tuberculosis infection. *Journal of Infectious Diseases*.

[14] Degiacomi G., Benjak A., Madacki J. (2017). Essentiality of mmpL3 and impact of its silencing on *Mycobacterium tuberculosis* gene expression. *Scientific Reports*.

[15] Jurado J. O., Alvarez I. B., Pasquinelli V. (2008). Programmed death (PD)-1:PD-ligand 1/PD-ligand 2 pathway inhibits T cell effector functions during human tuberculosis. *The Journal of Immunology*.

[16] Alvarez I. B., Pasquinelli V., Jurado J. O. (2010). Role played by the programmed death-1-programmed death ligand pathway during innate immunity againstMycobacterium tuberculosis. *The Journal of Infectious Diseases*.

[17] Potian J. A., Rafi W., Bhatt K., McBride A., Gause W. C., Salgame P. (2011). Preexisting helminth infection induces inhibition of innate pulmonary anti-tuberculosis defense by engaging the IL-4 receptor pathway. *The Journal of Experimental Medicine*.

[18] Cai Y., Yang Q., Tang Y. (2014). Increased complement C1q level marks active disease in human tuberculosis. *PLoS One*.

[19] Chen X., Zhou B., Li M. (2007). CD4+CD25+FoxP3+ regulatory T cells suppress *Mycobacterium tuberculosis* immunity in patients with active disease. *Clinical Immunology*.

[20] Irizarry R. A., Hobbs B., Collin F. (2003). Exploration, normalization, and summaries of high density oligonucleotide array probe level data. *Biostatistics*.

[21] Bolstad B. M., Irizarry R. A., Astrand M., Speed T. P. (2003). A comparison of normalization methods for high density oligonucleotide array data based on variance and bias. *Bioinformatics*.

[22] Smyth G. K. (2005). Limma: linear models for microarray data. *Bioinformatics & Computational Biology Solutions Using R & Bioconductor*.

[23] Kaufmann J., Schering A. G. (2014). *Analysis of Variance ANOVA*.

[24] Kolde R. (2015). *Pheatmap: Pretty Heatmaps, R Package Version 1.0.8.*.

[25] Langfelder R. b.P., Horvath S. (2008). WGCNA: an R package for weighted correlation network analysis. *Bmc Bioinformatics*.

[26] Yu G., Wang L.-G., Han Y., He Q.-Y. (2012). clusterProfiler: an R Package for comparing biological themes among gene clusters. *OMICS: A Journal of Integrative Biology*.

[27] Ashburner M., Ball C. A., Blake J. A. (2000). Gene Ontology: tool for the unification of biology. *Nature Genetics*.

[28] Ogata H. (2000). KEGG: Kyoto Encyclopedia of genes and Genomes. *Nucleic Acids Research*.

[29] Bogdan M. G., Ghosh J. K., Tokdar S. T. (2008). *A Comparison of the Benjamini-Hochberg Procedure with Some Bayesian Rules for Multiple Testing*.

[30] Mogga S. J., Mustafa T., Sviland L., Nilsen R. (2002). Increased Bcl-2 and reduced Bax expression in infected macrophages in slowly progressive primary murine *Mycobacterium tuberculosis* infection. *Scandinavian Journal of Immunology*.

[31] Karimi S., Mohammadi F., Mir Afsharieh S. A. (2005). High expression apoptotic proteins; P53, FAS, and BAX associated with down regulation BCL2 in tuberculosis granulomas: an immunohistochemistry study. *Applied Physics Letters*.

[32] Perskvist N., Long M., Stendahl O., Zheng L. (2002). *Mycobacterium tuberculosis* promotes apoptosis in human neutrophils by activating caspase-3 and altering expression of Bax/Bcl-xL via an oxygen-dependent pathway. *The Journal of Immunology*.

[33] Li G., Liu G., Song N. (2015). A novel recombinant BCG-expressing pro-apoptotic protein BAX enhances Th1 protective immune responses in mice. *Molecular Immunology*.

[34] Kuijpers T. W., Tool A. T. J., van der Bij I. (2016). Combined immunodeficiency with severe inflammation and allergy caused by ARPC1B deficiency. *Journal of Allergy & Clinical Immunology*.

[35] Mahla R. S. (2013). Sweeten PAMPs: role of sugar complexed PAMPs in innate immunity and vaccine biology. *Front Immunol*.

[36] Chávezgalán L., Ramon-Luing L., Carranza C., Garcia I., Sada-Ovalle I. (2017). Lipoarabinomannan decreases galectin-9 expression and tumor necrosis factor pathway in macrophages favoring *Mycobacterium tuberculosis* intracellular growth. *Frontiers in Immunology*.

[37] Gautam U. S., Mehra S., Ahsan M. H., Alvarez X., Niu T., Kaushal D. (2014). Role of TNF in the altered interaction of dormant *Mycobacterium tuberculosis* with host macrophages. *PLoS One*.

[38] Rajaram M. V. S., Ni B., Morris J. D. (2011). *Mycobacterium tuberculosis* lipomannan blocks TNF biosynthesis by regulating macrophage MAPK-activated protein kinase 2 (MK2) and microRNA miR-125b. *Proceedings of the National Academy of Sciences*.

[39] Kundu M., Pathak S. K., Kumawat K. (2009). A TNF- and c-Cbl-dependent FLIPS-degradation pathway and its function in Mycobacterium tuberculosis-induced macrophage apoptosis. *Nature Immunology*.

